# Influence of site, stand, and soil factors on sapling regeneration in typical *Quercus* Forests of Northern China

**DOI:** 10.3389/fpls.2026.1800142

**Published:** 2026-03-24

**Authors:** Guangshuang Duan, Yuxin Cheng, Yingshan Jin, Xuefan Hu, Lixia Liu, Yankui Wei, Fang Liang, Ziyi Wang

**Affiliations:** 1 School of Mathematics and Statistics, Xinyang Normal University, Xinyang, China; 2 Henan Provincial Center for Applied Mathematics, Xinyang, China; 3 Beijing Academy of Forestry and Landscape Architecture, Beijing, China; 4 Beijing Jingxi Forest Farm, Beijing, China; 5 Beijing Forestry and Parks Planning and Resource Monitoring Center, Beijing Forestry Carbon and International Cooperation Affairs Center, Beijing, China; 6 College of Forestry, Beijing Forestry University, Beijing, China

**Keywords:** environmental factors, natural regeneration, random forest, sapling density, soil factors

## Abstract

**Introduction:**

This study aims to characterize sapling regeneration in typical *Quercus* forests in Beijing, investigate the relationship between sapling density and environmental factors, and provide a theoretical basis for sustaining the ecological stability of *Quercus* forests in northern China.

**Methods:**

During June–September 2023, sapling density (individuals with diameter at breast height [DBH]1–5 cm) and environmental variables were surveyed across 17 plots in five *Quercus* forest types. Correlation analysis, random forest variable importance ranking, and multiple linear regression were used to identify key environmental factors affecting sapling density.

**Results:**

In *Q. mongolica* stands, sapling density (>1000 stems/ha) and mean DBH were the highest, while the stands of the other four *Quercus* species each maintained densities below 500 stems/ha with comparable DBH values. Sapling density was positively correlated with total stem number (N), canopy density (CD), and stand density (SD), but negatively with mean DBH (MD) and available copper (ACu). Random Forest analysis ranked the variables with relative importance exceeding 5% in the following order: MD > altitude (AL) > SD > Shannon-Wiener index (H´) > ACu > humus layer thickness (HLT) > N > CD. Multiple linear regression identified slope aspect, SD, and ACu as the primary environmental factors affecting sapling density.

**Discussion:**

Sapling abundance and growth status vary across *Quercus* forests in Beijing, with regeneration density co-regulated by multiple environmental factors. Therefore, forest management should integrate both biotic and abiotic factors to promote natural regeneration.

## 1 Introduction

Natural forest regeneration is a pivotal ecological process that sustains ecosystem services and dictates the trajectory of community succession and functional stability (Chazdon, 2014; Chazdon and Guariguata, 2016; Zhang et al., 2015). Against the backdrop of ongoing global environmental change, the evolution of adaptive traits determines a species’ regeneration potential by shaping its ability to complete critical life-history stages (Dey et al., 2019; Haq et al., 2022). Throughout all stages of the regeneration process, the survival and growth of seedlings and saplings serve as critical determinants of overall regeneration success. These factors define the future composition and structure of forest stands (Liu et al., 2022), laying the foundation for long-term sustainability.

The natural regeneration process is primarily influenced by site conditions (Lu et al., 2023; Redmond and Kelsey, 2018), stand characteristics (Ali et al., 2019; Li and Wang, 2023; Mölder et al., 2019), and soil factors (Annighöfer et al., 2015; Ma et al., 2023), while also being constrained by disturbance factors including human activities (e.g., hunting and forest fires) (Bharathi and Prasad, 2017; Dey et al., 2019; Hessburg et al., 2019). Site factors (such as altitude, slope gradient, aspect, and slope position) exert regulatory effects on regeneration density and spatial patterns by shaping local environmental variation (Albrecht and McCarthy, 2009; Li et al., 2012; Tiwari et al., 2020). For instance, sapling density showed a significant hump-shaped relationship with altitude, peaking at medium altitudes (approximately 1,900 m) and decreasing at both higher and lower elevations (Dani and Baniya, 2024). Stand factors (such as tree diversity, stand density, and canopy density) exert significant influence on forest regeneration by regulating the growth, development, and adaptive traits of saplings (Nilson and Lundqvist, 2001; Schweitzer and Dey, 2011; Vizoso-Arribe et al., 2014). Areas with high plant diversity promote community regeneration via niche differentiation, creating a multidimensional resource environment for trees at various developmental stages (e.g., seedlings, saplings, and mature trees) (Subashree et al., 2021). Soil factors (soil nutrients, soil physicochemical properties) jointly influence sapling stress resistance through nutrient supply and microbial interactions (Dyderski et al., 2018; Mölder et al., 2019). Furthermore, during forest regeneration, saplings represent an intermediate yet critical developmental stage, serving as the key transition from seedlings to mature trees (Wakeling et al., 2011). The survival status of saplings constitutes a multidimensional indicator, serving as a critical link in maintaining ecosystem function and stability (Sun et al., 2018; Thomas et al., 2016).

Natural regeneration is driven by a complex interplay of environmental factors. Correlation analysis, structural equation modeling, and linear regression are among the most widely used approaches to identify its key determinants. Dong et al. (2023) applied Pearson’s correlation analysis to investigate critical environmental factors affecting seedling density in the natural regeneration of *Q. mongolica* secondary forests. Yao et al. (2024) used structural equation modelling to conclude that undecomposed litter thickness directly affects the natural regeneration of *Larix principis-rupprechtii* monoculture plantations in northern China. Redmond and Kelsey (2018) employed linear regression to demonstrate a significant negative correlation between Engelmann spruce (*Picea engelmannii*) sapling density and altitude. Most existing studies on environmental controls of natural regeneration have been limited to either isolated analytical approaches or a narrow set of explanatory variables. Consequently, a systematic understanding of the combined influences of site, stand, and soil factors, and how these effects differ among forest types, remains lacking.

*Quercus* species are vital components of northern China’s regional forests (Dong et al., 2023; Zhang et al., 2019), forming climax communities through mixed stands with coniferous and broad-leaved species (Chen et al., 2003). They perform irreplaceable ecological functions in water conservation, soil preservation, and wildfire prevention (Huang et al., 2019). *Quercus* forests account for 11.95% of China’s total forest area and 9.98% of its total timber volume, representing a principal element of the nation’s natural woodlands (National Forestry and Grassland Administration, 2019). The mountainous regions of Beijing serve as a core distribution area for *Quercus* forests, harboring extant natural stands of *Quercus acutissima*, *Quercus mongolica*, *Quercus aliena*, *Quercus dentata*, and *Quercus variabilis* (Wu et al., 2004). Compared to artificial afforestation models, natural regeneration of *Quercus* species can reduce forestry management costs (Dey et al., 2019). However, under the combined effects of environmental stress and human disturbance, northern *Quercus* forests commonly face regeneration barriers, specifically manifested in the short survival cycle of saplings (Yu et al., 2017). Furthermore, a systematic research framework for sapling regeneration in northern *Quercus* forests is still lacking, particularly regarding the in-depth exploration of how multi-factor interactions influence sapling density (Huang et al., 2019; Wu et al., 2025). This study systematically analyzes the growth status and environmental drivers of saplings in Beijing’s typical Quercus forests to identify key limiting factors and provide a scientific basis for conservation.

This study focuses on *Quercus* forests in the Beijing region, with the primary research objectives being: (1) To analyze the quantitative characteristics and growth status of saplings within *Quercus* forests; (2) To employ correlation analysis to examine the influence of environmental factors on sapling density in *Quercus* forests; (3) Rank the importance of environmental factors affecting sapling density using the random forest algorithm; (4) Identify key environmental factors influencing sapling density through multiple linear regression analysis. The findings aim to provide theoretical foundations for the efficient utilization and management of *Quercus* forest resources in northern China, which will contribute to conserving biodiversity and maintaining ecosystem stability.

## 2 Materials and methods

### 2.1 Study area

Beijing (39°24′~41°36′N, 115°42′~117°24′E) lies in the northern part of the North China Plain, covering a total forest resources 853,100 hectares (Beijing Municipal Bureau of Statistics, 2024), with *Quercus* forests covering 122,700 hectares, accounting for 19.74% of the city’s total tree forest area. Elevations range from 20 to 1,500 m, with an average altitude of 43.5 m. Beijing exhibits a warm temperate, semi-humid continental monsoon climate. The city has an annual average temperature range of 10 - 13 °C and average annual precipitation of 500–600 mm, with a frost-free period lasting 180–200 days. Rainfall distribution is uneven, predominantly concentrated during summer. Predominant soil types include mountain brown earth, podzolic soils, and mountain meadow soils. The dominant vegetation within the study area comprises *Quercus acutissima*, *Quercus variabilis*, *Quercus mongolica*, *Quercus aliena*, *Quercus dentata*, and *Fraxinus chinensis*, accompanied by a few companion species such as *Acer truncatum*, *Carpinus turczaninowii*, and *Cotinus coggygria*.

### 2.2 Experiment design

**Figure 1:**
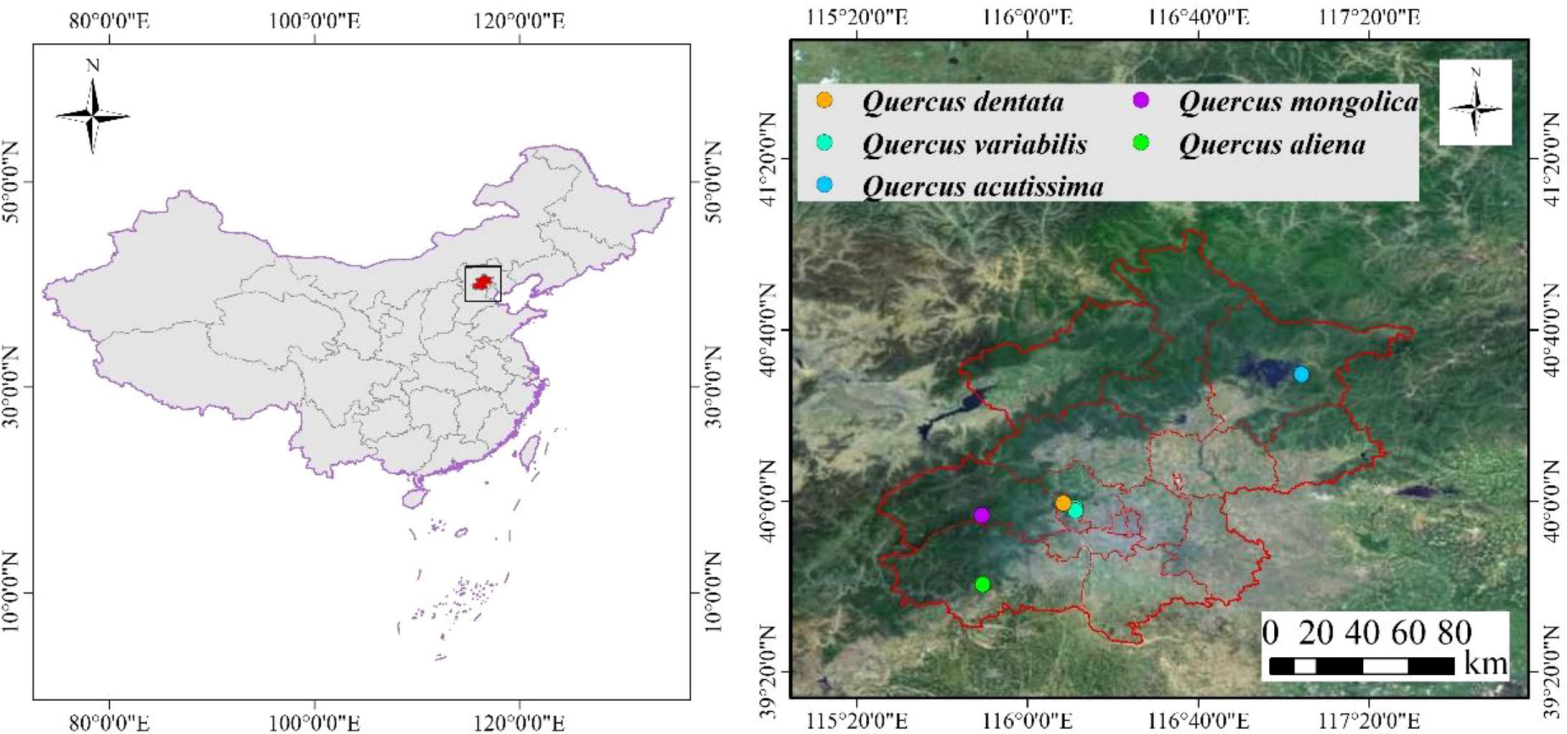
Location information for survey plots of five *Quercus* species in the Beijing region: *Q. dentata*, *Q. variabilis*, *Q. acutissima*, *Q. mongolica*, and *Q. aliena*. "Source: Adapted from Hu et al. (2025), published under CC BY 4.0.".

Field surveys of typical *Quercus* communities in Beijing were conducted from June to September 2023. Representative plots were selected within areas of concentrated *Quercus* distribution for study, encompassing five forest types. Plot locations are indicated in **Figure 1**.

A total of 17 square plots were established using a typical sampling method. Among them, four plots were set in both *Q. acutissima* and *Q. variabilis* stands, while three plots each were established in *Q. mongolica*, *Q. aliena*, and *Q. dentata* stands. The plot size ranged from the largest at 50 m × 50 m to the smallest at 20 m × 20 m. All trees within the plots with a diameter at breast height (DBH) exceeding 1 cm were individually measured. Records were compiled for each tree, including species name, number of trees, tree height, DBH, canopy density, and coordinates. The number and species composition of saplings with DBH between 1 and 5 cm were statistically analyzed. GPS was employed to determine plot elevation, aspect, slope position, and slope gradient. Aspect was categorized as north-facing (0°–135°, 225°–360°) or south-facing (135°–225°). Slope position was classified as upper, middle, or lower. Slope gradient was categorized as steep (>25°), gentle (15°–24°), or moderate (5°–14°). Soil samples from the 0–30 cm layer were collected using the profile method at the four corners and center point of the fixed plot to determine soil physicochemical properties. Five 1 m × 1 m sample plots were established near the five soil sampling plots to measure the thickness of the humus layer within each. Soil pH was measured using a pH meter, organic matter content was determined by the potassium dichromate method, total phosphorus by the molybdenum-antimony colorimetric method, and available copper by DTPA extraction followed by atomic absorption spectrophotometry (Bao, 2000). Species diversity in *Quercus* forests was assessed using the Shannon-Wiener index (H´) (Ma et al., 1995).

### 2.3 Index selection

To eliminate the effect of varying plot sizes on data comparability, all area-based raw measurements (e.g., density) were converted to a per-hectare basis prior to analysis. To explore the factors influencing sapling density in *Quercus* forests, a total of 14 environmental factors were selected based on literature review, multicollinearity diagnosis, and correlation analysis. These comprised 4 site factors (altitude, aspect, slope position, and slope gradient), 5 stand factors (Shannon-Wiener index, canopy density, stand density, mean DBH, and total stem number), and 5 soil factors (humus layer thickness, available copper, pH, organic matter, and total phosphorus). The data distribution characteristics for sapling density and influencing factors in *Quercus* stands are presented in **Table 1**.

**Table 1:** Environmental characteristics of study plots.

Factor Type	Units	Abbr.	Max	Min	Mean ± SD
Sapling Density	n/ha	Y	1883	75	496 ± 461
Altitude	m	AL	950	186	495.82 ± 291.70
Shannon-Wiener index	–	H´	2.34	1.09	1.48 ± 0.33
Canopy density	–	CD	0.90	0.65	0.78 ± 0.08
Stand density	n/ha	SD	2913.75	349.65	1200.17 ± 725.69
Mean DBH	cm	MD	25.68	7.74	16.42 ± 5.36
Total stem number	n	N	309	17	132 ± 86
Humus layer thickness	cm	HLT	11.00	2.00	4.88 ± 2.25
Available copper	mg/kg	ACu	1.75	0.47	1.04 ± 0.42
pH	–	pH	7.05	5.41	6.46 ± 0.48
Organic matter	g/kg	SOM	74.83	15.81	43.84 ± 16.45
Total phosphorus	g/kg	TP	0.95	0.18	0.42 ± 0.19

### 2.4 Statistical analysis

Using Excel to analyze the number characteristics and growth status of saplings in five *Quercus* stands. Secondly, Pearson correlation analysis was employed to examine the relationships between sapling density (Y) and site factors (altitude, aspect, slope position, and slope gradient), stand factors (Shannon-Wiener index, canopy density, stand density, mean diameter at breast height, and total stem number), and soil factors (humus layer thickness, available copper, pH, organic matter, and total phosphorus). Random forest analysis was performed using the randomForest package in R. The random forest analysis was performed with the following parameters: the number of trees (ntree) was set to 1000; the number of variables tried at each split (mtry) used the regression default of floor(p/3), where p is the number of predictor variables (p = 14 in this study, thus mtry = 4). Variable importance was evaluated based on the increase in node purity. Multiple linear regression models analyzed the relationship between sapling density and environmental factors, with optimal models obtained by backward stepwise regression. Model evaluation criteria comprised root mean square error (RMSE), coefficient of determination (R²), and total relative error (TRE). Correlation analysis, random forest algorithms, and multiple linear regression models were implemented using R v4.4.1 software (R Development Core Team, 2024). The correlation analysis was performed using the corrplot (Wei and Viliam, 2024) and GGally (Schloerke et al., 2024) packages. The random forest algorithm was implemented with the randomForest package (Liaw and Wiener, 2002). All graphics were generated using the ggplot2 (Wickham, 2016) and dplyr (Wickham et al., 2023) packages in R.

## 3 Results

### 3.1 Numerical characteristics and growth status of saplings in *Quercus* stands

**Figure 2:**
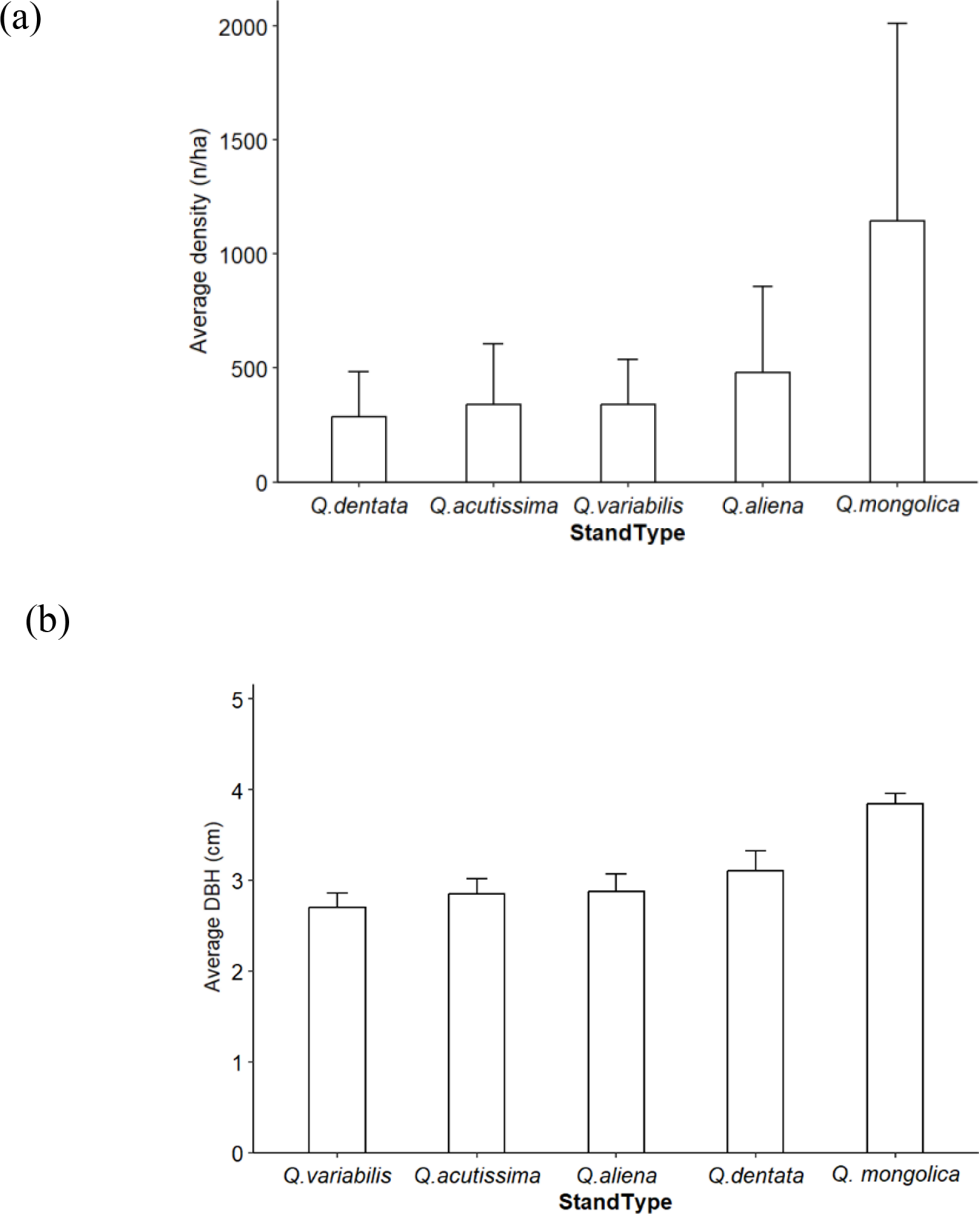
Characteristics of sapling indices in *Quercus* stands [**(a)** average density **(b)** average density DBH].

**Figure 2** illustrates the sapling density and average diameter at breast height for five *Quercus* stands. As shown in **Figure 2a**, the sapling densities of *Q. dentata*, *Q. acutissima*, *Q. variabilis*, *Q. aliena* stands were all below 500 stems/ha, compared with an average of over 1,000 stems/ha for *Q. mongolica* stands, marking the highest regeneration density. As shown in **Figure 2b**, the mean DBH of *Q. mongolica* stands was significantly larger than that of the other four *Quercus* stand types, among which no significant differences were found *Q. mongolica* stands had the largest mean DBH, indicating better sapling growth in this forest type. The mean DBH of saplings in *Q. acutissima*, *Q. aliena*, *Q. dentata*, and *Q. variabilis* stands was concentrated at or below 3 cm. In summary, with the exception of *Q. mongolica* stands, there were no significant differences in sapling density or mean DBH among *Q. acutissima*, *Q. aliena*, *Q. dentata*, and *Q. variabilis* stands.

### 3.2 Analysis of factors influencing sapling density in *Quercus* stands

#### 3.2.1 Correlation coefficients between sapling density and site factors in *Quercus* stands

**Figure 3:**
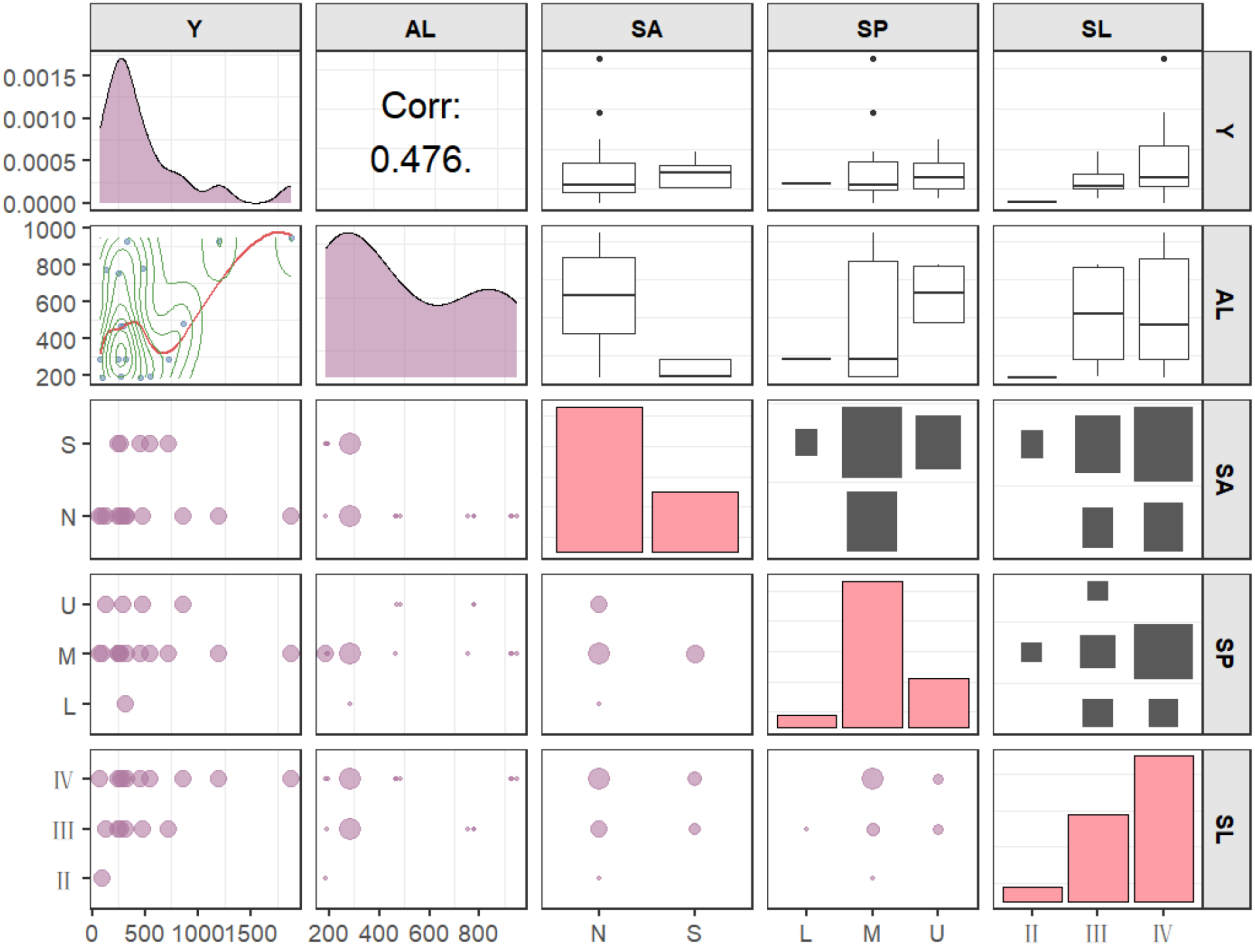
Pairwise relationship diagram between sapling density (Y) and four site factors (AL, Altitude; SA, Slope aspect; SP, Slope position; SL, Slope gradient; N, North-facing slope; S, South-facing slope; L, Upper slope position; M, Middle slope position; U, Lower slope position; II, gentle; III, moderate; IV, Steep).

Pairwise relationship plots between four site factors (AL, SA, SP, SL) and sapling density in *Quercus* stands (scatter plots for continuous variables, bubble plots for categorical variables) are shown in **Figure 3**. It is evident that sapling density exhibits an overall right-skewed distribution. Altitude shows a positive but non-significant correlation with sapling density; however, the scatter plot indicates that the rate of increase in sapling density slows at altitudes above 800m. Slope aspect, slope position, and slope gradient are three categorical variables. The bubble plot reveals no discernible association with sapling density.

#### 3.2.2 Correlation coefficients between sapling density and stand factors in *Quercus* stands

**Figure 4:**
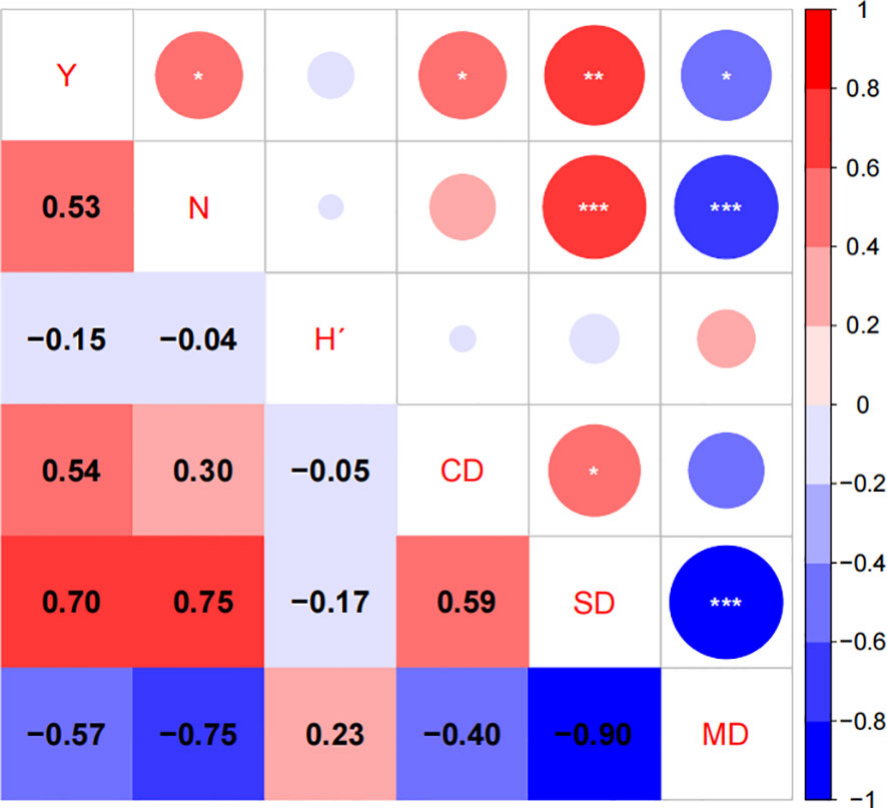
Heatmap of correlation coefficients between sapling density (Y) and stand factors (*p<0.05; **p<0.01; ***p<0.001; N: total number of trees; H´: Shannon-Wiener index; CD: canopy density; SD: stand density; MD: mean DBH).

The correlation coefficients and their significance levels between five stand factors (H´, CD, SD, mean DBH, and N) and sapling density in *Quercus* stands are presented in **Figure 4**. As shown in **Figure 4**, sapling density (Y) exhibits a significant positive correlation with total stem number (N), canopy density (CD), and stand density (SD). It shows a significant negative correlation with mean DBH (MD) and a non-significant negative correlation with the Shannon-Wiener index (H´). Total stem number (N) showed a significant positive correlation with stand density (SD) and a significant negative correlation with mean DBH (MD). Canopy density (CD) exhibited a significant positive correlation with stand density (SD). Stand density (SD) demonstrated a significant negative correlation with mean DBH (MD). With the exception of the Shannon-Wiener index (H´), all other stand factors exerted either direct or indirect influences on sapling density within *Quercus* stands.

#### 3.2.3 Correlation coefficients between sapling density and soil factors in *Quercus* stands

**Figure 5:**
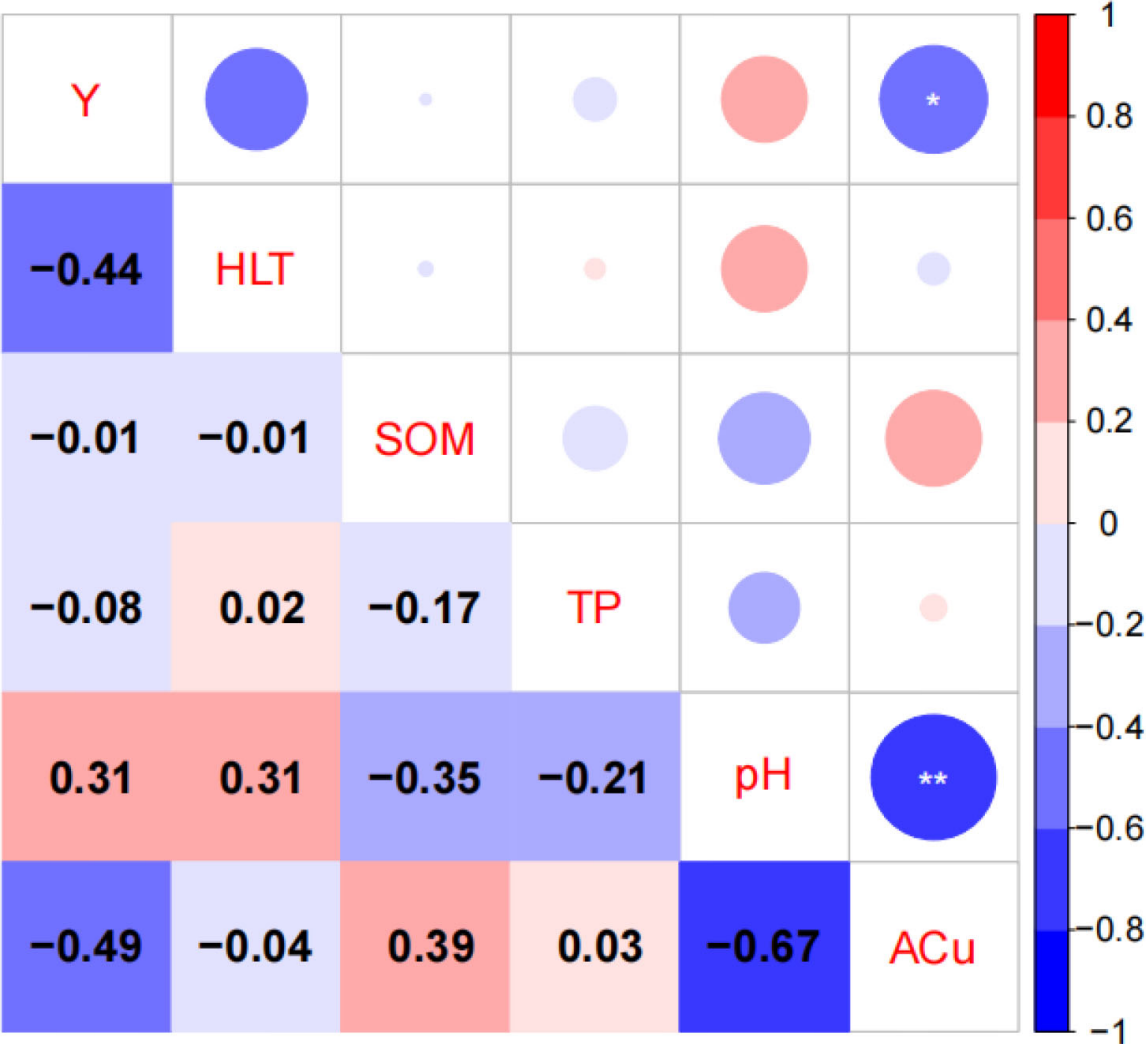
Heatmap of correlation coefficients between sapling density (Y) and soil factors (*p<0.05, **p<0.01; HLT, humus layer thickness; SOM, organic matter; TP, total phosphorus; pH, pH; ACu, available copper).

The correlation coefficients and significance levels between five soil factors (HLT, SOM, TP, pH, and ACu) and sapling density in *Quercus* stands are presented in **Figure 5**. As shown in **Figure 5**, sapling density (Y) was significantly and negatively correlated with ACu. As for the other soil factors, sapling density showed no significant correlations with HLT, SOM, or TP, and a non-significant positive correlation with pH. Additionally, pH was significantly negatively correlated with ACu. Beyond this, no other soil factors showed a significant correlation with sapling density.

#### 3.2.4 Ranking of key environmental factors for sapling density in *Quercus* stands

**Figure 6:**
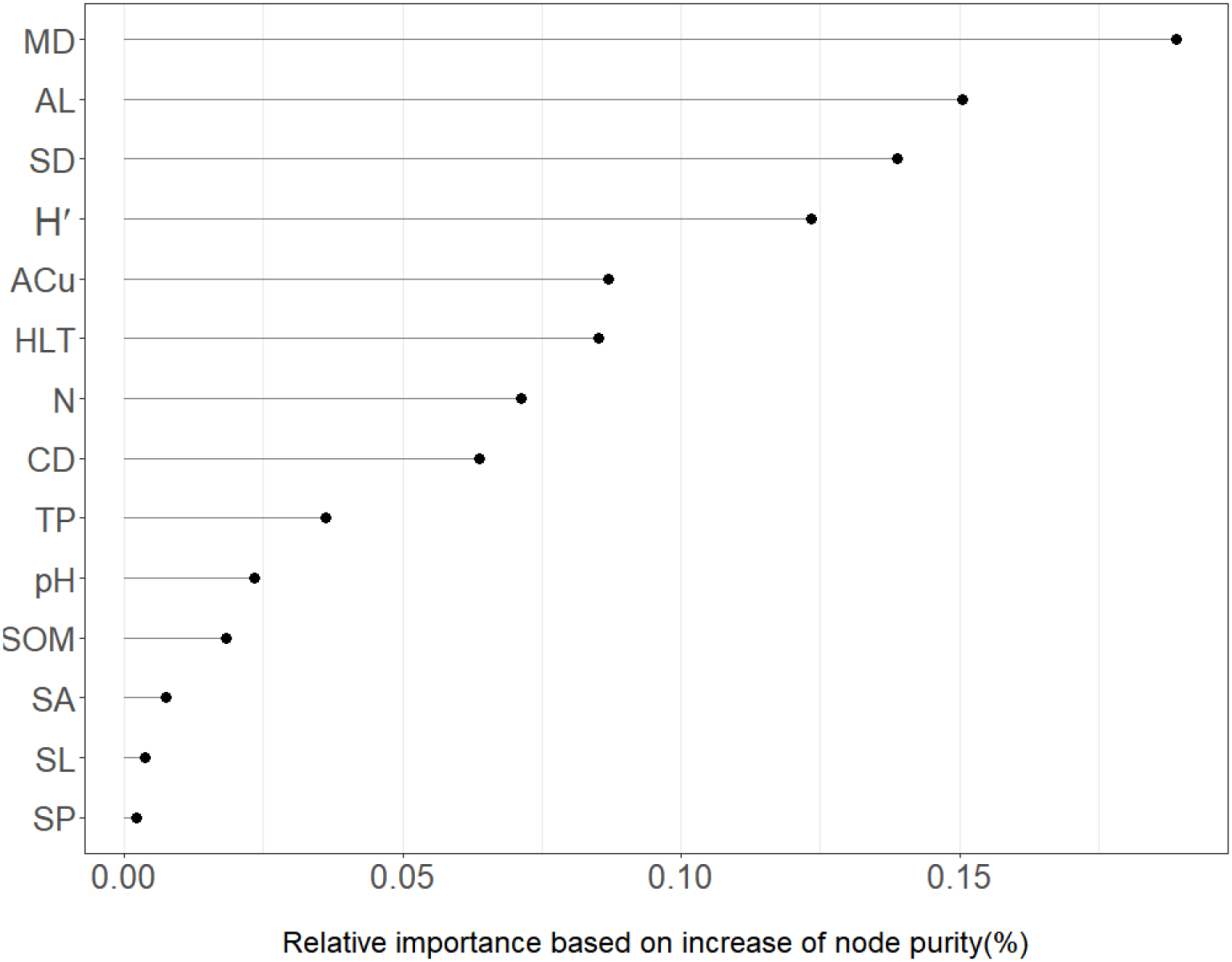
Relative importance of environmental factors for sapling density: A node purity increase analysis.

The results of the relative importance ranking between sapling density in *Quercus* stands and 14 environmental factors, based on the node purity improvement method using the random forest algorithm, are shown in **Figure 6**. Environmental factors with relative importance exceeding 5% were ranked as follows: MD > AL > SD > H´ > ACu > HLT > N > CD. This sequence included one site factor, five stand factors, and two soil factors. In summary, sapling density in *Quercus* stands is driven by multiple environmental factors, with stand and soil factors playing a pivotal role.

### 3.3 Analysis of environmental factors affecting sapling density based on multiple linear regression models

Based on the results of the correlation analysis, a multiple linear regression model was established with sapling density as the dependent variable and CD, SD, MD, and ACu as independent variables. Collinearity diagnostics showed that the Variance Inflation Factor (VIF) values for SD and ACu in the final model were both 1.16 (VIF<5), indicating no serious multicollinearity concerns. The optimal model (Model (1), **Table 2**) had an RMSE of 298.47, an R² of 0.55, and a TRE of 24.94%. Similarly, based on the ranking of important factors from the random forest analysis, a multiple linear regression model was established with sapling density as the dependent variable and MD, SD, AL, H´, HLT, and ACu as independent variables. The VIF values for AL, SD, and ACu in the final model were 3.79, 3.87, and 1.61, respectively (VIF < 5), indicating no serious multicollinearity. The optimal model (Model (2), **Table 2**) had an RMSE of 275.50, an R² of 0.62, and a TRE of 20.49%. In summary, Model (2) demonstrated satisfactory predictive accuracy and explanatory power.

**Table 2:** Multivariate linear regression model fitting results for sapling density in *Quercus* stands.

Model	Multiple linear regression model formulation	Performance metric		
		RMSE	R²	TRE
(1)	y=353.93+0.38SD−302.69ACu	298.47	0.55	24.94
(2)	y=440.89+0.65SD−319.76ACu−0.79AL	275.50	0.62	20.49

S, Sapling density; SD, Stand density; ACu, Available copper; AL, Altitude.

The fitting results of Model (1) and Model (2) indicate that SD, AL, and ACu significantly influence sapling density, with ACu exerting a greater effect. However, Model (2) demonstrates superior predictive performance for sapling density compared to Model (1).

## 4 Discussion

### 4.1 Numerical characteristics and growth status of saplings in *Quercus* stands

This study indicates that among the five *Quercus* stands surveyed in the Beijing region, no significant differences were observed in in sapling quantitative characteristics or growth indicators, except within the *Q. mongolica* stand. This phenomenon can be explained by regional environmental factors and stand structure. On the one hand, Beijing’s climate features cold, dry winters and summers with concentrated precipitation and significant interannual variability, coupled with susceptibility to drought in spring and autumn (Yang et al., 2023). These environmental conditions pose severe challenges to sapling survival and growth. On the other hand, *Quercus* stands within the study area are predominantly in the middle-aged to mature stage, exhibiting high canopy density that significantly reduces understory light penetration and results in resource scarcity, particularly light availability (Zhou et al., 2026). In summary, the combined effects of these stand factors define the suitability of understory microhabitats, which in turn determines the growth potential of saplings.

### 4.2 Analysis of factors influencing sapling density in *Quercus* stands

#### 4.2.1 Sapling density and site factors

Research indicates that site factors (altitude, aspect, slope position, and slope gradient) exert no significant influence on sapling density within *Quercus* stands. This contrasts with findings by Ren et al. (2019), who observed that aspect and slope gradient significantly affected tree regeneration density in *Q. acutissima* stands. This discrepancy may stem from differences in species diversity and composition across forest types, as well as environmental variations between southern and northern regions. This study found that among the site factors influencing sapling density, altitude ranked higher in relative importance than other factors. This may be related to the thermal gradient indirectly driven by altitude: for instance, the decline in mean annual temperature with increasing elevation can increase the sensitivity of saplings to low temperatures, thereby reducing their survival rates at higher elevations (Tiwari et al., 2020; Vera, 1997). Alternatively, the limited number of plots and the small number of site factor levels involved may have resulted in insignificant effects.

#### 4.2.2 Sapling density and stand factors

This study reveals significant associations between forest stand factors and sapling density within forest communities. Sapling density exhibits significant positive correlations with total stem number, canopy density, and stand density, while showing a negative correlation with mean DBH. This indicates that denser stands composed of smaller trees offer more favorable conditions for natural regeneration. Total stem number and stand density promote the population size of parent trees, enhancing seedling survival rates through seed dispersal and thereby increasing sapling density in understory regeneration (Boyden et al., 2005). Some studies have reported that canopy density promotes sapling density (Huang and Liu, 2018; Huang et al., 2019; Kang et al., 2011), which aligns with our findings. High canopy closure primarily affects sapling regeneration by altering the understory’s light, temperature, and humidity conditions. Moderate shading can reduce surface evaporation and maintain soil moisture. Additionally, the buffering effect of the forest canopy mitigates diurnal and seasonal temperature variations, providing a relatively mild and stable microenvironment conducive to the photosynthesis, growth, and development of saplings. However, mean DBH inhibits sapling density; larger trees may monopolize resources, leading to understory resource scarcity and thereby suppressing light-demanding species. The significant positive correlation among stand density, total stem number, and canopy density reflects interdependent ecological processes. As stand density increases, intraspecific competition leads to uneven resource allocation (light, water, and nutrients), inducing saplings to compete for canopy space through vertical growth strategies. This process ultimately results in a significant reduction in sapling mean DBH (Ali et al., 2019).

This study further observed a non-significant negative correlation between the Shannon-Wiener index and sapling density, though the importance ranking indicated approximately 12% relative importance. Existing research demonstrates a significant positive influence of plant species diversity indices on natural stand regeneration (Poorter et al., 2021; Ren et al., 2019), contrasting with these findings. This discrepancy may stem partly from dominant tree species monopolizing resources and outcompeting others through rapid growth, thereby reducing species diversity. Alternatively, the impact of species diversity may exhibit a lag effect, requiring several years of succession to manifest. In summary, the combined influence of these stand factors shapes the suitability of understory microhabitats, thereby determining the growth potential of saplings.

#### 4.2.3 Sapling density and soil factors

This study found a significant negative correlation between sapling density and available copper, whilst soil pH also exhibited a significant negative correlation with available copper. Among soil factors, available copper and humus layer thickness ranked highly in relative importance, indicating key interactions between soil physicochemical properties and forest regeneration dynamics. Available copper within soil nutrients significantly influenced sapling density, with similar findings corroborated in prior studies on other community regenerations (Li et al., 2021). Copper in the soils of the Beijing mountainous region originates from both natural and anthropogenic sources, including the local soil parent material, atmospheric deposition, and historical local mining (Zheng et al., 2005; Zhuo et al., 2019). Copper constitutes an essential component of plant antioxidant enzymes. Available copper enhances sapling disease resistance, promotes nutrient uptake, and optimizes soil conditions. However, available copper inhibits root development, suppresses soil microbial activity, and disrupts nutrient absorption, thereby increasing seedling mortality and reducing sapling recruitment density – a pattern consistent with findings in warm-temperate forests (Zhao et al., 2015). The interaction between soil pH and copper concentration jointly influences fungal and bacterial growth in soil. Elevated soil pH mitigates copper toxicity (Fernández-Calviño and Bååth, 2016), a mechanism consistent with the findings of this study.

This study further found no significant correlation between sapling density and humus layer thickness, total phosphorus, or organic matter content. However, humus layer thickness ranked relatively high in importance (above 5%), potentially because sapling growth in *Quercus* forests depends more on other factors (such as light, soil moisture, and temperature) (Dyderski et al., 2018; Liu et al., 2022). The five *Quercus* stands exhibited broad pH tolerance; across the measured range, pH did not significantly affect sapling recruitment density. In summary, the key soil factor limiting forest regeneration in the study area is the availability of copper, which exerts a significant inhibitory effect on sapling recruitment density. Soil nutrients, including humus layer thickness and organic matter content, showed no significant direct regulatory influence.

### 4.3 Analysis of multiple linear regression model results

Analysis of the multiple linear regression model indicates that the key environmental factors influencing sapling density are stand density, available copper, and altitude. Among these, available copper exhibits the highest absolute value of the standardized regression coefficient, demonstrating strong explanatory power within the model and representing the primary driver of sapling density variation. The two multiple linear regression models constructed using different sources of independent variables exhibited differing explanatory capacities for sapling density variation, accounting for 55% and 62% of the variation respectively. The remaining unexplained variation may stem from unmeasured climatic factors and anthropogenic disturbances.

### 4.4 Limitations

The relatively limited number of sample plots in this study may have reduced its statistical power, potentially hindering the detection of significant effects from environmental factors with subtle influences or complex mechanisms. Additionally, the factors influencing natural regeneration are diverse and intricate. Other elements, such as forest gaps, light conditions, and disturbances, can substantially affect stand regeneration as well. However, due to survey constraints, data on only a subset of these factors was obtained. More importantly, this study is based on data from a single survey period. Because forest succession is a long-term dynamic process, the mechanisms suggested by our findings require further investigation through long-term monitoring.

## 5 Conclusions

Among five *Quercus* stands in the Beijing region, no significant differences in sapling regeneration characteristics were observed between stands other than *Q. mongolica*. The primary environmental factors influencing sapling density in Beijing’s *Quercus* stands include site factors (altitude), stand factors (total stem number, Shannon-Wiener index, canopy density, stand density, mean DBH), and soil factors (available copper). Altitude is the most significant site factor affecting sapling density, while total stem number, Shannon-Wiener index, canopy density, stand density, and mean DBH are the principal stand factors. Available copper is the most critical soil factor influencing sapling density. Therefore, in the management of *Quercus* stands, it is recommended to establish core conservation zones in the mid-to-low altitude areas surveyed in this study (186–950 m). For higher altitude areas, planting cold-tolerant oak species may be considered, although their suitability requires further investigation. Concurrently, targeted thinning should be employed to increase canopy openings and optimize light distribution. Additionally, appropriate liming to raise soil pH can reduce copper ion activity. Forest management must also holistically consider biotic and abiotic factors to overcome resource limitations and promote natural regeneration.

## Data Availability

The original contributions presented in the study are included in the article/supplementary material. Further inquiries can be directed to the corresponding author/s.
